# Combination of Radiomics Features and Functional Radiosensitivity Enhances Prediction of Acute Pulmonary Toxicity in a Prospective Validation Cohort of Patients with a Locally Advanced Lung Cancer Treated with VMAT-Radiotherapy

**DOI:** 10.3390/jpm12111926

**Published:** 2022-11-18

**Authors:** Vincent Bourbonne, François Lucia, Vincent Jaouen, Olivier Pradier, Dimitris Visvikis, Ulrike Schick

**Affiliations:** 1 Radiation Oncology Department, University Hospital, 29200 Brest, France; 2 LaTIM, INSERM, UMR 1101, University of Western Brittany, 29200 Brest, France; 3 Institut Mines-Télécom Atlantique, 29200 Brest, France

**Keywords:** radiation pneumonitis, lung cancer, prediction, cluster of voxel, radiomics, personalized medicine

## Abstract

**Simple Summary:**

Despite strict application of dose constraints, acute pulmonary toxicity (APT) remains frequent, and may impact treatment compliance and patient quality of life. Prediction models based on either a radiomics approach or a voxel-based approach were previously developed, but never combined. Combination of radiomics features and functional radiosensitivity enhances prediction of acute pulmonary toxicity. Correction of imbalanced data enhances prediction of APT. Use of such prediction models opens the possibility of tailored dosimetry planning.

**Abstract:**

Introduction: The standard of care for people with locally advanced lung cancer (LALC) who cannot be operated on is (chemo)-radiation. Despite the application of dose constraints, acute pulmonary toxicity (APT) still often occurs. Prediction of APT is of paramount importance for the development of innovative therapeutic combinations. The two models were previously individually created. With success, the Rad-model incorporated six radiomics functions. After additional validation in prospective cohorts, a Pmap-model was created by identifying a specific region of the right posterior lung and incorporating several clinical and dosimetric parameters. To create and test a novel model to forecast the risk of APT in two cohorts receiving volumetric arctherapy radiotherapy (VMAT), we aimed to include all the variables in this study. Methods: In the training cohort, we retrospectively included all patients treated by VMAT for LALC at one institution between 2015 and 2018. APT was assessed according to the CTCAE v4.0 scale. Usual clinical and dosimetric features, as well as the mean dose to the pre-defined Pmap zone (DMean_Pmap_), were processed using a neural network approach and subsequently validated on an observational prospective cohort. The model was evaluated using the area under the curve (AUC) and balanced accuracy (Bacc). Results: 165 and 42 patients were enrolled in the training and test cohorts, with APT rates of 22.4 and 19.1%, respectively. The AUCs for the Rad and Pmap models in the validation cohort were 0.83 and 0.81, respectively, whereas the AUC for the combined model (Comb-model) was 0.90. The Bacc for the Rad, Pmap, and Comb models in the validation cohort were respectively 78.7, 82.4, and 89.7%. Conclusion: The accuracy of prediction models were increased by combining radiomics, DMean_Pmap_, and common clinical and dosimetric features. The use of this model may improve the evaluation of APT risk and provide access to novel therapeutic alternatives, such as dose escalation or creative therapy combinations.

## 1. Introduction

In inoperable patients, radiotherapy (RT) combined with chemotherapy (Ch) is the standard of care in stage III lung cancer [1], according to international guidelines [2]. Modern radiation techniques, such as intensity-modulated radiotherapy (IMRT) and volumetric-arctherapy (VMAT), enable a higher conformation, resulting in lower doses to organs at risk (OARs) without compromising coverage of the planning target volume (PTV). Despite these advances, acute pulmonary toxicity (APT) remains frequent with a 5–25% rate [3]. 

APT appears to be significantly correlated to higher doses delivered to the lungs and possibly to the heart. However, strict application of organ-based dose constraints appear insufficient for prevention of such toxicities. This may be explained by the fact that dose volume histograms (DVH) do not efficiently account for spatial dose distribution or organ architecture. Radiomic features could better apprehend dose distribution heterogeneity [4] and, when applied to dose maps, appear as efficient predictors of APT [5]. A second explanation could be the lack of functional organ-based dose constraints. For this purpose, several techniques were developed for the identification of such functional sub-regions [6,7,8,9]. Most of these techniques require performing costly nuclear imaging, such as ^68^Ga PET-CT or Ventilation-Perfusion SPECT-CT, and raise multiple questions regarding inter-imaging modality registration and guidelines for the definition of functional volumes. Based on retrospective data, a voxel-based analysis approach selected voxels significantly associated with the APT endpoint, defining a significance map [10,11,12]. In patients treated with VMAT-radiotherapy for lung cancer, a volume was identified in the posterior right lung. Higher doses to this volume (Pmap) were associated with a significantly higher risk of APT. Despite robust results, this model suffered from overfitting with loss of performance in the testing cohort. The radiomics model was only internally validated, and prospective evaluation remains to be tested. Furthermore, the possibility of the combination between the radiomics and Pmap approaches was never tested, nor was its interest evaluated. Given the relatively low rate of grade ≥2 APT (20–25%), the combination of the two approaches could lead to a higher performance, but only in the training cohort, due to overfitting.

In this study, we aim to combine the radiomics and voxel-based approaches and develop a new model that enhances the prediction of APT in patients treated for a locally advanced lung cancer treated with a VMAT-based RT.

## 2. Materials and Methods

### 2.1. Population

A previously presented population was used, consisting of 165 retrospectively and 42 prospectively included patients [12]. All patients were treated with curative intent for a histologically-proven locally advanced lung cancer (non-small cell or small-cell lung cancer) between 2015 and 2020. Patients with aged <18 years old and follow-up less than 1 year after RT completion were excluded. Similarly, patients with a history of thoracic radiation therapy, pneumonectomy, or incomplete RT were excluded. When performed, Ch could be delivered as a sequential or concomitant treatment. RT was delivered with a prescription dose of 60 to 66 Gy to the PTV, with 95% of the dose covering 95% of the prescription volume. All RT courses were delivered with VMAT. The study was approved by the hospital ethical committee (Retrospective cohort: NCT04545658, Prospective cohort: NCT03931356).

### 2.2. Toxicities

Using the CTCAE v4.0, acute pulmonary toxicity was graded as follows: APT3 was defined as a binary outcome relating to a grade 3 pulmonary toxicity event happening during the first 6 months after the start of RT, whereas APT2 was defined as a binary outcome related to a grade 2 pulmonary toxicity event occurring during the first 6 months after the start of RT. APT was retrospectively gathered for every patient in the retrospective cohort. APT was measured and prospectively collected as a secondary outcome for the prospective cohort. Actuarial incidences were employed.

### 2.3. Clinical and Dosimetric Features

Age, gender, performance status (PS), administration of Ch, tumor stage as determined by the American Joint Committee on Cancer (AJCC), mean expiratory volume/second (MEVS), history of smoking, and chronic obstructive pulmonary disease were all gathered as standard clinical features (COPD).

Regarding dosimetric features, all usual dose constraints for the homolateral lung (LungH), contralateral lung (LungC), both lungs (Lungs) and the heart were considered. The full list of dosimetric features is available as Supplementary Table S1.

### 2.4. Radiomics and Pmap Features

When combined in the radiomics-based model (Rad-model), six radiomic features were previously identified as highly correlated with the risk of APT_2_ [5]: the Variance (LungH_Variance), Difference Variance (LungH_DVAR), Contrast (LungH_Contrast), and Entropy (LungH_Entropy), extracted from the LungH volume based on the Co-occurence Matrix, as well as the IC1 Information measure of correlation (LungH_IC1) extracted from the LungH volume, and the Energy extracted from the lung volume and based on the histogram (Lungs_Energy). All radiomics features were extracted using the in-house IBSI-compliant software [13] (MIRAS©), with a fixed bin number of 1 (1 Gy = 1 grey-level).

A region (Pmap) was previously identified in the posterior right lung [12]. Combining the mean dose to this Pmap region (DMean_Pmap_) with 10 other features, a Pmap-model was built, strongly associated with the risk of APT ≥ grade 2. All features used for the Pmap-model [12] were thus considered for the model building and optimization.

### 2.5. Statistical Analysis

The overall population was considered as two separate and independent cohorts: the training cohort, regrouping the 165 retrospectively evaluated patients, and the testing cohort, consisting of the 42 prospectively included patients, as previously presented [12]. Feature set selection and model building were developed on the training cohort, blinded from the testing set. Each model was then evaluated on the testing cohort. Several prediction models were separately built using a neural network (NN) approach. Based on this NN approach, the radiomics and Pmap models were previously presented using respectively 6 [5] and 11 [12] features. Given the fact that the Rad-model was developed on a subset of the actual training cohort, the Rad-model was retrained. The Pmap-model was tested as previously developed.

All clinical, dosimetric, and radiomics features were considered for the development of the Combined model (Comb-model), using the same NN approach. The Multilayer Perceptron is a tool embedded in SPSS Modeler v18.0, producing a prediction model based on the APT endpoint and ranking each feature by its importance. To reduce the number of included features, features were combined using a decremental approach; at each step, the least important feature is deleted, and the model is retrained until only one feature remains. For every combination, the model was stabilized using a bootstrap approach with n = 1000 replications. For each set of features, only the model with the highest mean accuracy based on the 1000 replications was retained. A probability threshold was defined as the value maximizing the Youden Index defined by

$$YI=Sensitivity\;\left(Sp\right)+Specificity\;\left(Se\right)-1.$$

Given the low rate of APT_2_ and risk of unbalanced data, the impact of a correction using oversampling via the Synthetic Minority Oversampling Technique (SMOTE) package was evaluated [14]. Six prediction models were thus developed:

- Three without SMOTE: Rad_NonSmote_, Pmap_NonSmote_, and Comb_NonSmote_
- Three with SMOTE: Rad_Smote_, Pmap_Smote_, and Comb_Smote_

The performance of each model was then analyzed using sensitivity (Se), specificity (Sp), balanced accuracy (Bacc: mean of the Se and Sp), F1-score, and positive (PPV) and negative (NPV) prediction values in both the training and testing cohorts. Decision curves analysis (DCA) for the training and testing sets was performed plotting the net benefit with its corresponding threshold. Precision-recall curves were also used for model comparisons.

Given the clinical importance of Grade 3 APT (APT_3_), a secondary analysis was performed for the prediction of APT_3_. New cut-offs were defined on the training cohort, maximizing the Youden Index as presented, but for the prediction of APT_3_.

In the event of radiomic features being retained in the Combined models, an analysis of its regional significance was planned. For practicality, only the most important radiomic feature retained in the Comb_Smote_ model was analyzed. Three-dimensional radiomics maps were calculated for each patient based on the lung volume, using the Pyradiomics toolbox [15], with a kernel set to 3. We then used the same methodology as previously used for the definition of the Pmap-region. The protocol was previously fully detailed [10,12]. Briefly, radiomics maps were registered to a thoracic phantom using a segment-based registration. A voxel-based analysis was then performed, with n = 10.000 replications, using a *t*-test approach evaluating the significance of each voxel for differentiating patients with APT_2_ from the patients without APT_2_. Correction for multiple testing was used (family-wise error). The significance map based on the most important radiomic feature retained in the Comb_Smote_ model was named: PmapRad. Overlap between the Pmap and PmapRad maps was evaluated using the DICE coefficient.

## 3. Results

### 3.1. Population

The population characteristics have been previously presented [12,16]. For completeness, patient characteristics according to the occurrence of APT_2_ in each cohort are summarized in Supplementary Table S2. The rate of APT ≥ grade 2 was 22.4 and 19.1% in the training and testing cohorts, respectively, whereas only 11 patients presented with an APT ≥ grade 3 (9 in the training and 2 in the testing sets, respectively).

### 3.2. Radiomics and Pmap-Models

**Figure 1:**
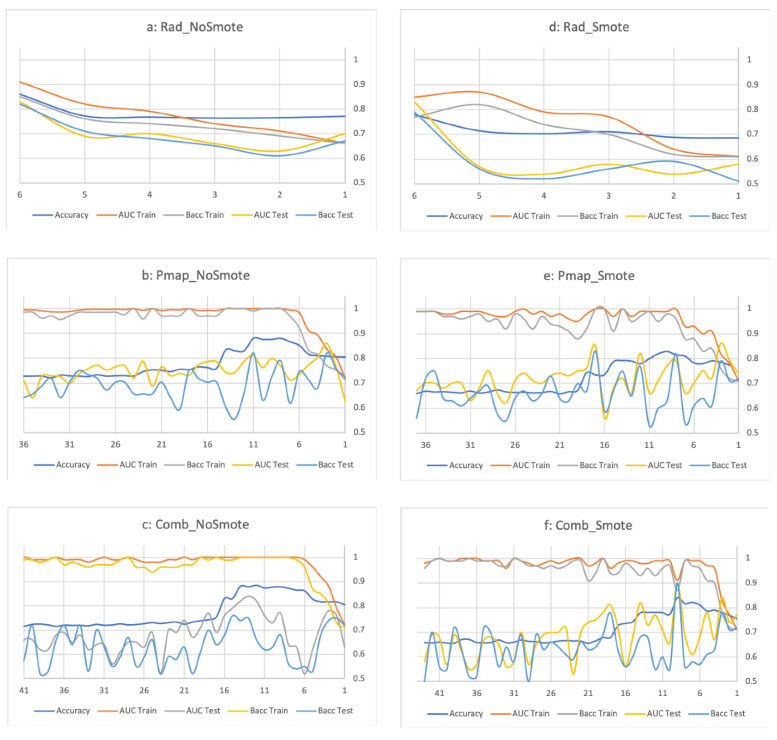
Performance of each model (Rad_NoSmote_ (**a**), Rad_Smote_ (**b**), Pmap_NoSmote_ (**c**), Pmap_Smot_ (**d**), Comb_NoSmote_ (**e**), and Comb_Smote_ (**f**)) according to the number of retained features for the prediction of APT ≥ grade 2. Abbreviation: Accuracy: Mean accuracy based on the bootstrap n = 1000 replications, APT: Acute pulmonary toxicity, Rad_NoSmote: Radiomics model without Smote, Rad_Smote: Radiomics model with Smote, Pmap_NoSmote: Pmap model without Smote, Pmap_Smote: Pmap model with Smote, Comb_NoSmote: Combined model without Smote, Comb_Smote: Combined model with Smote. Legend: x-axis: number of features in the model, y-axis: mean accuracy (n = 1000 bootstrap replications) of the model.

The retrained Rad_NonSmote_ and Rad_Smote_ models achieved AUCs of 0.91/0.85 in the training cohort (Table 1) and 0.83/0.83 in the testing cohort (Table 2). Performance of the Rad_NonSmote_ and Rad_Smote_ models, according to the number of retained features, is available as Figure 1a,b, respectively, with the chosen model combining the six radiomic features. Ranking of the six included radiomics features was different between the two radiomics models. The radiomics models were obtained combining the six radiomic features, the most important feature being the Energy_Hist_Lungs_, accounting for 58.5% of the NN-model (Table 3), whereas Variance_Cooc_LungH_ ranked as the most important (43.0%) for the Rad_Smote_ model. In the testing cohort, Baccs were 82.0/78.7% with 18/24.0% probability thresholds for the Rad_NonSmote_ and Rad_Smote_, respectively (Table 2).

The Pmap_NonSmote_ model was previously presented [12] and resulted in an AUC of 1.00 and 0.81 in the training and testing sets, respectively. Consequently, the deducted Baccs were 99.2% in the training set (Table 1) and 82.0% in the testing set (Table 2). Adding a correction of unbalanced data using the SMOTE approach, the Pmap_Smote_ model combined eight features and resulted in a Bacc of 82.0% in the testing cohort (6% threshold). Performance of the Pmap_NonSmote_ and Pmap_Smote_ models, according to the number of retained features, is available as Figure 1c,d, respectively.

As planned, 3D radiomics maps were extracted for each patient, using the LungH_Variance feature. Overlap between the PmapRad and Pmap maps is presented as Supplementary Figure S1 and appears to be relatively low (DICE coefficient of 0.35).

### 3.3. Combined Model

For the combined models, 43 features were considered and their correlation with the risk of APT_2_ was evaluated (Supplementary Table S3). Without SMOTE, the best model achieved a mean accuracy of 0.88 (Figure 1e) and combined 12 different features (Table 3). In this model (Comb_NonSmote_), the three most important features were the DMean_Pmap_, Dmean_2Lungs_, and Variance_Cooc_LungH_, with respective importances of 32.7, 10.1, and 7.1%. On the training cohort, the Comb_NonSmote_ model achieved an AUC of 0.91 and a Bacc of 86.2% when applying a 8% threshold (Table 1). On the testing cohort, this model resulted in a Bacc of 69.5%. Correct classification occurred for 89.7% of low-risk and 38.5% of high-risk patients (Table 2).

Use of the SMOTE approach resulted in the development of a different combined model (Figure 1f), the Comb_Smote_ model, combining nine features, with the three most important features remaining the same: DMean_Pmap_, Variance_Cooc_LungH_, and Dmean_2Lungs_, with respective importances of 44.1, 12.6, and 10.5% (Table 3). In the testing cohort, the Comb_Smote_ model resulted in a Bacc of 89.7% using the 12% threshold, with the highest PPV (53.3%) while maintaining a 100% NPV.

### 3.4. Model Comparison for the Prediction of APT ≥ Grade 2 and APT ≥ Grade 3

**Figure 2:**
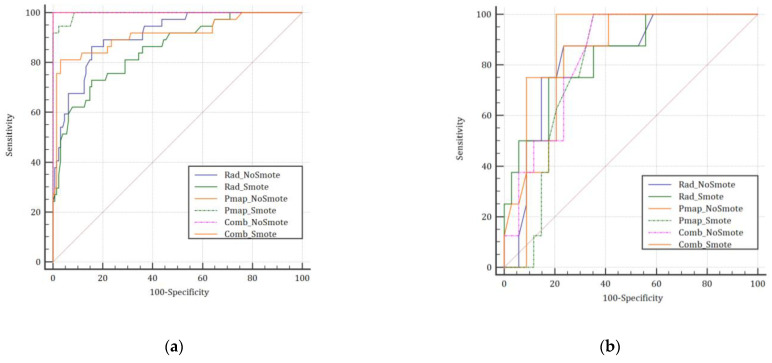
Comparison between each model in the training (**a**) and testing (**b**) sets based on the ROC curve for the prediction of APT ≥ grade 2. Abbreviation: Rad_NoSmote: Radiomics model without Smote, Rad_Smote: Radiomics model with Smote, Pmap_NoSmote: Pmap model without Smote, Pmap_Smote: Pmap model with Smote, Comb_NoSmote: Combined model without Smote, Comb_Smote: Combined model with Smote.

For APT_2_, the Pmap_NoSmote_, Pmap_Smote_, and Comb_NoSmote_ achieved the highest results based on the curves in the training cohort (Figure 2a), whereas no significant differences were observed between the six models in the testing cohort (Figure 2b).

**Figure 3:**
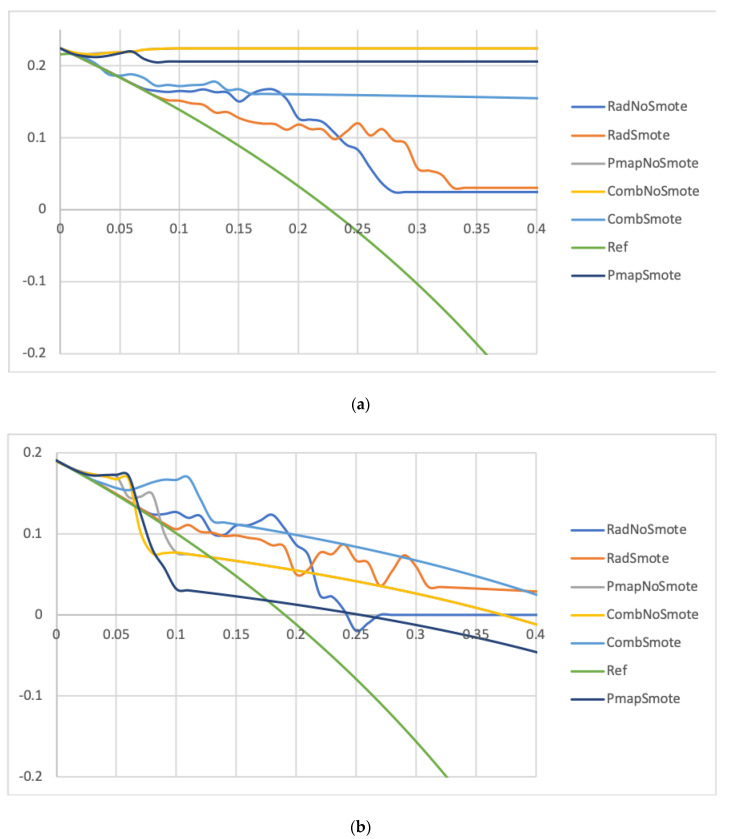
Comparison between each model in the training (**a**) and testing (**b**) sets based on the decision curve analysis for the prediction of APT ≥ grade 2. Abbreviation: APT: Acute pulmonary toxicity, Rad_NoSmote: Radiomics model without Smote, Rad_Smote: Radiomics model with Smote, Pmap_NoSmote: Pmap model without Smote, Pmap_Smote: Pmap model with Smote, Comb_NoSmote: Combined model without Smote, Comb_Smote: Combined model with Smote. Legend: x-axis: Probability threshold, y-axis: Net clinical benefit.

Though both the six-prediction models achieved interesting results in the testing set, the Comb_Smote_ model achieved better results than the Comb_NoSmote_, Rad, and Pmap models for the overall range of predicted probabilities (Figure 3). Similarly, the F1 score for the Comb_Smote_ model surpassed all other models in the testing cohort (0.71 vs. 0.56–0.63). Precision-recall and calibration curves favored Smote-based models, except for the Pmap models (Supplementary Figures S2 and S3, respectively).

In both cohorts, according to the DCA, the radiomics model achieved the lowest clinical benefit for predicted probabilities >20%. On the other hand, for predicted probabilities <20%, net clinical benefit seemed to overlap in the testing cohort.

Regarding the prediction of APT ≥ grade 3 in the training cohort, three prediction models ranked first (Pmap_NoSmote_, Pmap_Smote_, and Comb_NoSmote_), with a Bacc of 0.92 and newly defined respective cut-offs (Supplementary Table S4, Supplementary Figure S4a). On the testing cohort and with a 86% threshold, the Pmap_Smote_ model ranked first, with a Bacc of 0.94, a NPV of 100%, but a low PPV of 28.6% (Supplementary Table S5, Supplementary Figure S4b). Apart from the Rad_NoSmote_, all remaining models performed similarly with Baccs ranging from 0.85 to 0.93 and a NPV of 100.0% for all. Based on the DCA, the Pmap_Smote_ and Comb_NoSmote_ models harbored a favorable profile in the training cohort (Supplementary Figure S5a), whereas the Pmap_Smote_ stood out in the testing cohort (Supplementary Figure S5b). To be noted, clinical benefits remained relatively small for all prediction models.

## 4. Discussion

Stage III lung cancer is a unique setting, where a tailored approach regarding response to treatment and risk of adverse events is mandatory. Indeed, though immunotherapy (IT) has changed the landscape of patients with locally advanced lung cancer [3,17], access to these adjuvant treatments must be increased. Furthermore, several phase II and phase III trials are currently ongoing to evaluate the benefit of IT with Ch-RT. In a recent Phase II trial [18] evaluating two different Ch-IT combinations with RT, 67 out of 112 patients treated with carboplatin—paclitaxel—pembrolizumab (patients with non-squamous or squamous lung cancer) discontinued treatment, 62.2% because of adverse events. Though objective response rates were interesting (approximately 70% in each arm), radiation pneumonitis of any grade was observed in 17.9% of the patients. Technical features regarding dosimetry planning and dose–volume histograms for both the PTV and OARs were insufficiently detailed in prospective trials evaluating treatments in this setting. Several studies assessing the compliance of the delivered planning to the protocols proved that survival was positively impacted by both volume definition and dose constraints [19,20]. One might think the initial dosimetry planning could also impact patients’ tolerance to treatment. Given the number of trials evaluating Ch-IT with RT combinations [18,21,22,23,24,25,26], we developed two different approaches.

Our first approach considered the impact of spatial dosimetry and heterogeneity of the dose received by both lungs [5]. In the testing cohort, the Rad_NoSmote_ model retrained on 165 patients achieved a lower Bacc than previously reported with a Bacc of 82.0% in the testing set. Based on only six radiomics features, this model is easily applicable but can only be considered as a method for evaluating dosimetry plans.

With a different approach based on voxel-based analysis, a cluster of voxels sensitive to radiotherapy was localized in the posterior right lung. Combining 11 features, the Pmap_NoSmote_ model achieved similar results on the testing set (Bacc of 82.0%) compared with the radiomics model. Interestingly, the DCA favored the Pmap model for an APT_2_ predicted probability superior to 20%. On the contrary, for probabilities inferior to 20%, performances of the two models overlapped. These two approaches are relatively innovative but have never been combined.

In the combined approach, we considered 12 features, among which the DMean_Pmap_ and a radiomics feature (LungH_Variance) were associated. The Comb_NoSmote_ model was associated with an AUC of 0.82 and Bacc of 69.5%, thus a lower Bacc mainly due to lower sensitivity. Addition of the SMOTE approach enhanced the performance of three newly developed prediction models. With a NPV of 100%, the rate of false positives decreased from 53.3% for the Rad_NoSmote_ and Pmap_NoSmote_ models to 46.7%. This model consisting of a two-step approach probably better apprehended the complexity of dose delivery to a functionally heterogenous organ such as the lung. The first step, consisting of an evaluation of the DMean_Pmap_ and other clinical and dosimetric features, offers the possibility of adaptative planning with a goal of decreasing the DMean_Pmap_. Once the dosimetry planning is completed, radiomics features are extracted and combined with previous features to produce a risk probability. A few advantages were found, including better results for APT_2_ and APT_3_ prediction. On the other hand, this approach remains more complex and less easily implemented for a clinical use. External validation of our models is currently under investigation. Despite validation in a prospectively recruited cohort, multicentric validation is needed. Several factors, such as treatment delivery heterogeneity, toxicity evaluation, and racial and ethnic differences, as well as psychosocial factors, which could not be assessed in our cohorts, should be added to fully evaluate model performances.

Apart from the Rad_Smote_ and Comb_Smote_, all models were prone to overfitting with significant performance loss between training and testing cohorts. Rad_Smote_ and Comb_Smote_ achieved harbored correction for imbalanced data that seemed to reduce this limitation and should be considered in future prediction models when dealing with skewed endpoints. The Comb_Smote_ model also stood out from the other models with the highest F1 score.

Having more efficient APT prediction tools could greatly benefit patients undergoing RT for lung cancer. Patients at high predicted risk of APT should be considered for dosimetry optimization until an acceptable risk is reached. When unfeasible, careful monitoring under treatment or neoadjuvant chemotherapy could be proposed. Patients at low risk of APT could benefit from dose escalation or treatment combination, with the addition of IT during CT-RT followed by adjuvant IT, as studied in several phase III trials [18,21,22,23,24,25,26].

Main alternatives to such machine-learning approaches are based on nuclear imaging and require costly exams, such as ventilation (V)/perfusion (P), SPECT-CT, or ^68^Ga PET-CT [6,7,8]. These approaches have the advantage of offering the possibility of adaptative planning, based on the physiological structure of the lungs. However, several technical issues arise, such as 4D inter-modality registration and variability of the definitions of functional and non-functional lungs. Furthermore, the possibility of effective optimization of dosimetry planning must be demonstrated. Such approaches could further enhance prediction modeling and treatment planning.

Though the Pmap and PmapRad volumes appeared complementary, both regions were mainly encompassed in the right lung, confirming the specific role of these sub-regions regarding the risk of APT. Despite an overlap between these regions and functional regions, as defined through nuclear imaging [6], the precise physiopathology underlying the role of these regions needs further research. Specific vascularization of the posterior right lung could be a primary explanation. Sensitivity of these regions to low-dose volumes (V5_LungH_ and V10_LungH_) and their impacts on respiratory functions, especially on the diffusion capacity of the lungs for carbon monoxide (DLCO), could partly explain our results. A phase 2 study (NCT04942275) being conducted at our institution focuses on patients treated with stereotactic radiotherapy (SBRT), aimed at better understanding regional radiation sensitivity [27]. Despite it being on a different population, substantial information will be extracted from this study and may explain the specific role of the posterior right lung.

Despite its few limitations, this study brings new insight for prediction modelling in patients treated with radiotherapy and marks the final step before clinical validation in a randomized setting. With two competitive models, comparison and combination appear necessary to choose the best model for validation. This choice is based on prediction efficiency, but also generalizability, ease of use, and integration in the usual workflow, with the Pmap model being the most balanced model. This manuscript stands out from previous studies due to several factors. Due to the relatively moderate cohort and high number of features, a correction of imbalanced data was performed, enhancing the overall results for several models. As hypothetized, all non-SMOTE models suffered from overfitting. This was the main benefit of the SMOTE approach, with enhanced performance for the Comb_Smote_ model regarding the prediction of grade > 2 APT. Finally, this work brought a focus on APT_3_ prediction with, again, the combination model reaching the highest performances.

## 5. Conclusions

The accuracy of the APT2 prediction models is increased by combining radiomics, DMeanPmap, and common clinical and dosimetric features. The combined model appears to be more effective for the prediction of APT ≥ grades 2 and 3 than other prediction models, such as the Pmap-model in patients treated with VMAT for locally advanced lung cancer. These two models are useful tools for clinicians to reduce the risk of APT in selected patients and are ready for prospective and external validation in a multicentric setting. Following this validation, a randomized study will be initiated comparing outcomes in patients treated with dosimetric optimization based on the previously presented Pmap model and patients treated without dosimetric optimization. Such a randomized trial will need a simple, efficient, and generalizable prediction model that would be either embedded in the treatment planning system or act as a standalone tool. Furthermore, the use of this model may provide access to novel therapeutic alternatives, such as dose escalation or creative therapy combinations.

## Figures and Tables

**Table 1:** Analysis of each model’s discrimination between patients with or without APT ≥ grade 2 in the training set.

Set	AUC	*p*	Cut-Off	C-Index	Se	Sp	BAcc	F1	Number of Patients, n (%)					
									Below the Cutoff (Low Risk of APT ≥ Grade 2)			Above the Cutoff (High Risk of APT ≥ Grade 2)		
									Total	Without APT	With APT	Total	Without APT	With APT
Rad_NoSmote_	0.91	<0.0001	>18%	0.85	86.5	84.4	85.5	0.72	113 (68.5%)	108 (95.6%)	5 (4.4%)	52 (31.5%)	20 (38.5%)	32 (61.5%)
Rad_Smote_	0.85	<0.0001	>24%	0.77	75.7	78.1	76.9	0.66	109 (66.1%)	100 (91.7%)	9 (8.3%)	56 (33.9%)	28 (50.0%)	28 (50.0%)
Pmap_NoSmote_	0.99	<0.0001	>8%	0.99	100.0	98.4	99.2	1.00	126 (76.4%)	126 (100.0%)	0 (0.0%)	39 (23.6%)	2 (5.1%)	37 (94.9%)
Pmap_Smote_	0.99	<0.0001	>6%	0.96	100.0	91.4	95.7	0.96	117 (70.9%)	117 (100.0%)	0 (0.0%)	48 (29.1%)	11 (22.9%)	37 (77.1%)
Comb_NoSmote_	0.99	<0.0001	>8%	0.99	100	98.4	99.2	1.00	126 (76.4%)	126 (100.0%)	0 (0.0%)	39 (23.6%)	2 (5.1%)	37 (94.9%)
Comb_Smote_	0.91	<0.0001	>12%	0.87	81.1	92.2	86.7	0.85	125 (75.8%)	118 (94.4%)	7 (5.6%)	40 (24.2%)	10 (25.0%)	30 (75.0%)

Abbreviations: AUC: Area under the curve, Se: Sensitivity, Sp: Specificity, BAcc: Balanced Accuracy, F1: F1-score, APT: Acute pulmonary toxicity, Rad_NoSmote: Radiomics model without Smote, Rad_Smote: Radiomics model with Smote, Pmap_NoSmote: Pmap model without Smote, Pmap_Smote: Pmap model with Smote, Comb_NoSmote: Combined model without Smote, Comb_Smote: Combined model with Smote.

**Table 2:** Analysis of each model’s discrimination between patients with or without APT ≥ grade 2 in the testing set.

Set	AUC	*p*	Cut-Off	C-Index	Se	Sp	BAcc	F1	Number of Patients, n (%)					
									Below the Cutoff (Low Risk of APT ≥ Grade 2			Above the Cutoff (High Risk of APT ≥ Grade 2)		
									Total	Without APT	With APT	Total	Without APT	With APT
Rad_NoSmote_	0.83	<0.0001	>18%	0.82	87.5	76.5	82.0	0.63	27 (64.3%)	26 (96.3%)	1 (3.7%)	15 (35.7%)	8 (53.3%)	7 (46.7%)
Rad_Smote_	0.83	<0.0001	>24%	0.79	75.0	82.4	78.7	0.60	30 (71.4%)	28 (93.3%)	2 (6.7%)	12 (28.6%)	6 (50.0%)	6 (50.0%)
Pmap_NoSmote_	0.81	<0.0001	>8%	0.82	87.5	76.5	82.0	0.61	27 (64.3%)	26 (96.3%)	1 (3.7%)	15 (35.7%)	8 (53.3%)	7 (46.7%)
Pmap_Smote_	0.79	<0.0001	>6%	0.82	100.0	64.7	82.4	0.57	22 (52.4%)	22 (100.0%)	0 (0.0%)	20 (47.6%)	12 (60.0%)	8 (40.0%)
Comb_NoSmote_	0.83	<0.0001	> 8%	0.70	62.5	76.5	69.5	0.57	29 (69.0%)	26 (89.7%)	3 (10.3%)	13 (31.0%)	8 (61.5%)	5 (38.5%)
Comb_Smote_	0.90	<0.0001	>12%	0.90	100.0	79.4	89.7	0.71	27 (64.3%)	27 (100.0%)	0 (0.0%)	15 (35.7%)	7 (46.7%)	8 (53.3%)

Abbreviations: AUC: Area under the curve, Se: Sensitivity, Sp: Specificity, BAcc: Balanced accuracy, F1: F1 score, APT: Acute pulmonary toxicity, Rad_NoSmote: Radiomics model without Smote, Rad_Smote: Radiomics model with Smote, Pmap_NoSmote: Pmap model without Smote, Pmap_Smote: Pmap model with Smote, Comb_NoSmote: Combined model without Smote, Comb_Smote: Combined model with Smote.

**Table 3:** Classification of each feature by importance for each model.

Rad_NoSmote_ Model		Rad_Smote_ Model		Pmap_NoSmote_ Model		Pmap_Smote_ Model		Comb_NoSmote_ Model		Comb_Smote_ Model	
Feature	Importance	Feature	Importance	Feature	Importance	Feature	Importance	Feature	Importance	Feature	Importance
LungH_Variance	0.1%	LungH_Entropy	1.9%	V40_Heart_	5.0%	Stage	3.6%	V40_Heart_	4.4%	V13_LungH_	3.3%
LungH_DVAR	1.5%	Lungs_Energy	4.5%	DMean_LungH_	5.0%	V5_LungH_	5.0%	DMean_LungH_	5.0%	V5_LungH_	3.8%
LungH_Contrast	3.2%	LungH_IC1	15.6%	V5_LungH_	5.0%	MEVS	5.4%	V5_LungH_	5.0%	V10_LungH_	4.4%
LungH_IC1	15.2%	LungH_DVAR	16.9%	AJCC Stage	5.0%	V10_LungH_	6.1%	AJCC Stage	5.1%	DMean_LungH_	4.8%
LungH_Entropy	20.5%	LungH_Contrast	18.1%	V10_LungH_	6.0%	DMean_LungH_	6.6%	V10_LungH_	5.5%	V30_2Lungs_	6.3%
Lungs_Energy	58.5%	LungH_Variance	43.0%	COPD	6.0%	V30_2Lungs_	10.2%	COPD	6.1%	COPD	10.4%
				MEVS	7.0%	DMean_2Lungs_	15.5%	MEVS	6.1%	DMean_2Lungs_	10.5%
				Smoking Status	7.0%	DMean_Pmap_	47.7%	Smoking Status	6.2%	LungH_Variance	12.6%
				V30_2Lungs_	7.0%			V30_2Lungs_	6.7%	DMean_Pmap_	44.1%
				DMean_2Lungs_	11.0%			LungH_Variance	7.1%		
				DMean_Pmap_	36.0%			DMean_2Lungs_	10.1%		
								DMean_Pmap_	32.7%		

Abbreviations: V40_Heart_: Volume of the heart receiving 40 Gy, DMean_LungH_: Mean dose received by the homolateral lung, V5_LungH_: Volume of the homolateral lung receiving 5 Gy, AJCC Stage: American Joint Committee on Cancer, V10_LungH_: Volume of the homolateral lung receiving 10 Gy, COPD: Chronic obstructive pulmonary disease, MEVS: Mean expiratory volume/second, V30_2Lungs_: Volume of the 2 lungs receiving 30 Gy, DMean_2Lungs_: Mean dose received by the 2 lungs, DMean_Pmap_: Mean dose received by the Pmap volume, LungH: Homolateral lung, Lungs: both lungs, LungH_Variance: Variance extracted from the LungH volume on the Co-occurence matrix (Cooc), LungH_DVAR: Difference variance extracted from the LungH volume on the Cooc matrix, LungH_Contrast: Contrast extracted from the LungH volume on the Cooc matrix, LungH_IC1: Information measure of correlation extracted from the LungH volume on the Cooc matrix, LungH_Entropy: Entropy extracted from the LungH volume on the Cooc matrix, LungH, Lungs_Energy: Entropy extracted from the LungH volume on the histogram. Abbreviation: Rad_NoSmote: Radiomics model without Smote, Rad_Smote: Radiomics model with Smote, Pmap_NoSmote: Pmap model without Smote, Pmap_Smote: Pmap model with Smote, Comb_NoSmote: Combined model without Smote, Comb_Smote: Combined model with Smote.

## Data Availability

Access to data can be requested upon demand.

## Supplementary Materials

The following supporting information can be downloaded at: https://www.mdpi.com/article/10.3390/jpm12111926/s1, Supplementary Table S1: List of all studied dosimetric features, Supplementary Table S2: Main patient characteristics in the training and testing sets, Supplementary Table S3: Correlation of each feature with the APT_2_ risk (training cohort), Supplementary Table S4: Analysis of the each model’s discrimination between patients with or without APT ≥ grade 3 in the training set, Supplementary Table S5: Analysis of the each model’s discrimination between patients with or without APT ≥ grade 3 in the testing set, Supplementary Figure S1: Overlap between the PmapRad and Pmap maps, Supplementary Figure S2: Comparison between each model in the testing sets based on the precision-recall curve for the prediction of APT ≥ grade 2, Supplementary Figure S3: Comparison between each model in the testing sets based on the calibration curve for the prediction of APT ≥ grade 2, Supplementary Figure S4: Comparison between each model in the training (a) and testing (b) sets, based on the ROC curve for the prediction of APT ≥ grade 3, Supplementary Figure S5: Comparison between each model in the training (a) and testing (b) sets, based on the decision curve analysis for the prediction of APT ≥ grade 3.
