# Supplementary material for: Combination of Radiomics Features and Functional Radiosensitivity Enhances Prediction of Acute Pulmonary Toxicity in a Prospective Validation Cohort of Patients with a Locally Advanced Lung Cancer Treated with VMAT-Radiotherapy
Source: J Pers Med. 2022 Nov 18;12(11):1926. doi: 10.3390/jpm12111926 (PMC9693103; doi:10.3390/jpm12111926)
Supplement: Supplementary file 1 [file jpm-12-01926-s001.zip › jpm-1936427-supplementary.pdf]

SUPPLEMENTARY MATERIALS

Supplementary Table 1: List of all studied dosimetric features

Supplementary Table 2: Main patients' characteristics in the training and testing sets

Supplementary Table 3: Correlation of each feature with the APT_2 risk (training cohort)

Supplementary Table 4: Analysis of the each model's discrimination between patients with or without APT ≥ grade 3 in the training set

Supplementary Table 5: Analysis of the each model's discrimination between patients with or without APT ≥ grade 3 in the testing set

Supplementary Figure 1: Overlap between the PmapRad and the Pmap maps

Supplementary Figure 2: Comparison between each model in the testing sets based on the precision-recall curve for the prediction of APT ≥ grade 2

Supplementary Figure 3: Comparison between each model in the testing sets based on the calibration curve for the prediction of APT ≥ grade 2

Supplementary Figure 4: Comparison between each model in the training (a) and testing (b) sets based on the ROC curve for the prediction of APT ≥ grade 3

Supplementary Figure 5: Comparison between each model in the training (a) and testing (b) sets based on the Decision Curve Analysis for the prediction of APT ≥ grade 3

Supplementary Table 1: List of all studied dosimetric features

Total Dose RT
Vol_{GTV}
Vol_{CTV}
Vol_{PTV}
$DMean_{Heart}$
$V30_{Heart}$
$V40_{Heart}$
$DMean_{2Lungs}$
$V13_{2Lungs}$
$V20_{2Lungs}$
$V30_{2Lungs}$
$DMax_{LungH}$
$DMean_{LungH}$
$V5_{LungH}$
$V10_{LungH}$
$V13_{LungH}$
$V20_{LungH}$
$V30_{LungH}$
$DMax_{LungC}$
$DMean_{LungC}$
$V5_{LungC}$
$V10_{LungC}$
$V13_{LungC}$
$V20_{LungC}$
$V30_{LungC}$

Abbreviations: Vol: volume (mL), GTV: Gross Tumour Volume, CTV: Clinical Tumour Volume, PTV: Planning Tumour Volume, RT: Radiotherapy, $DMean_y$: Mean Dose received by the y volume, $DMax_y$: Maximum Dose received by the y volume, Vx_y: Volume of the y volume receiving x Gy, LungH: Homolateral Lung, LungC: Controlateral Lung, 2Lungs: both lungs

Supplementary Table 2: Main patients' characteristics in the training and testing sets

	Training cohort n = 165		Testing cohort n = 42		*p*
**Age mean** (SD)	65.0 (9.38)		65.7 (10.03)		0.67
**Gender**					
Male (nb, %)	111	67.3	31	73.8	0.42
Female (nb, %)	54	32.7	11	26.2	
**Median PS** (range)	1	0-2	1	0-2	0.06
**Smoking**					
Activ (nb, %)	62	37.6	17	40.5	0.52
Former/never (nb, %)	103	62.4	25	59.5	
**Known COPD** (nb, %)	63 (38.2)		12 (28.6)		0.25
**Mean MEVS** (%, CI 95%)	74.4 (71.1-77.6)		72.3 (65.7-78.9)		0.58
**Histology**					
SCC (nb, %)	61	37.0	16	38.1	0.79
ADC (nb, %)	67	40.6	17	40.5	
SCLC (nb, %)	24	14.5	6	14.3	
Others (nb, %)	13	7.9	3	7.1	
**AJCC stage** (Median)	IIIA		IIIA		0.70
**Total RT Dose**					
Median (Gy, range)	66.0 (54.0-66.0)		66.0 (54.0-66.0)		0.59
**Chemotherapy sequence**					
Concomitant (nb, %)	53	32.1	14	33.3	0.99
Induction (nb, %)	48	29.1	12	28.6	
Induction + concomitant (nb, %)	34	20.6	7	16.7	
None (nb, %)	30	18.2	9	21.4	
**Adjuvant durvalumab** (nb, %)	16	9.7	3	9.5	0.78
**APT rate (%)**	22.4		19.1		0.64

Abbreviations: SD: Standard Deviation, nb: number, %: percentage, COPD: chronic obstructive pulmonary disease, MEVS: mean expiratory volume/second, SCC: squamous-cell carcinoma, ADC: adenocarcinoma, SCLC: small-cell lung cancer, AJCC: American Joint Commission on Cancer, RT: radiotherapy, APT: Acute pulmonary toxicity ≥ grade 2

Supplementary Table 3: Correlation of each feature with the APT risk (training cohort)

**Feature**	**AUC**	***p***
Age	0.52	*0.70*
Gender	0.52	*0.66*
COPD	0.57	*0.15*
MEVS	0.59	*0.09*
Smoking status	0.57	*0.13*
AJCC Stage	0.53	*0.63*
Concomittant CT	0.53	*0.56*
Neoadjuvant CT	0.52	*0.71*
Neoadjuvant -> Concomittant CT	0.53	*0.38*
RT alone	0.50	*1.00*
Performance Status	0.55	*0.27*
Total Dose RT	0.51	*0.82*
Vol_{GTV}	0.62	***0.03***
Vol_{CTV}	0.61	***0.02***
Vol_{PTV}	0.62	***0.01***
$DMean_{Heart}$	0.52	*0.63*
$V30_{Heart}$	0.51	*0.79*
$V40_{Heart}$	0.54	*0.43*
$DMean_{2Lungs}$	0.60	***0.04***
$V13_{2Lungs}$	0.56	*0.25*
$V20_{2Lungs}$	0.56	*0.21*
$V30_{2Lungs}$	0.59	*0.07*
$DMax_{LungH}$	0.51	*0.86*
$DMean_{LungH}$	0.60	***0.04***
$V5_{LungH}$	0.57	*0.17*
$V10_{LungH}$	0.58	*0.13*
$V13_{LungH}$	0.58	*0.13*
$V20_{LungH}$	0.58	*0.12*
$V30_{LungH}$	0.59	*0.06*
$DMax_{LungC}$	0.53	*0.61*
$DMean_{LungC}$	0.51	*0.84*
$V5_{LungC}$	0.52	*0.78*
$V10_{LungC}$	0.53	*0.65*
$V13_{LungC}$	0.50	*0.98*
$V20_{LungC}$	0.53	*0.61*
$V30_{LungC}$	0.54	*0.49*
$DMean_{Pmap}$	0.69	***0.0005***
LungH_Variance	0.61	***0.04***
LungH_DVAR	0.53	*0.55*
LungH_Contrast	0.53	*0.57*
LungH_IC1	0.50	*0.96*
LungH_Entropy	0.52	*0.65*
Lungs_Energy	0.52	*0.79*

Abbreviations: COPD: Chronic Obstructive Pulmonary Disease, MEVS: mean expiratory volume/second, AJCC: American Joint Committee on Cancer, CT: Chemotherapy, GTV: Gross Tumour Volume, CTV: Clinical Tumour Volume, PTV: Planning Tumour Volume, RT: Radiotherapy, Vx_y: Volume of the y volume receiving x Gy, $DMean_y$: Mean Dose received by the y volume, $DMax_y$: Maximum Dose received by the y volume, LungH: homolateral lung, Lungs: both lungs, LungH_Variance: Variance extracted from the LungH volume on the Coocurence matrix (Cooc), LungH_DVAR: Difference Variance extracted from the LungH volume on the Cooc matrix, LungH_Contrast: Contrast extracted from the LungH volume on the Cooc matrix, LungH_IC1: Information measure of correlation extracted from the LungH volume on the Cooc matrix, LungH_Entropy: Entropy extracted from the LungH volume on the Cooc matrix, LungH, Lungs_Energy: Entropy extracted from the LungH volume on the Histogram.

Supplementary Table 4: Analysis of the each model's discrimination between patients with or without APT ≥ grade 3 in the training set

Set	AUC	p	Cut-off	C-index	Se	Sp	BAcc	Number of patients, n (%)					
								Below the cutoff (Low risk of APT ≥ grade 3)			Above the cutoff (High risk of APT ≥ grade 3)		
								Total	Without APT	With APT	Total	Without APT	With APT
$Rad_{NoSmote}$	0.90	< 0.0001	> 19%	0.87	100.0	74.4	87.2	116 (70.3%)	116 (100.0%)	0 (0.0%)	49 (29.7%)	40 (81.6%)	9 (18.4%)
Rad_{Smote}	0.82	< 0.0001	> 25%	0.82	88.9	75.0	82.0	118 (71.5%)	117 (99.2%)	1 (0.8%)	47 (28.5%)	39 (83.0%)	8 (17.0%)
$Pmap_{NoSmote}$	0.92	< 0.0001	> 89%	0.92	100.0	84.6	92.3	132 (80.0%)	132 (100.0%)	0 (0.0%)	33 (20.0%)	24 (72.7%)	9 (27.3%)
$Pmap_{Smote}$	0.91	< 0.0001	> 86%	0.92	100.0	84.6	92.3	132 (80.0%)	132 (100.0%)	0 (0.0%)	33 (20.0%)	24 (72.7%)	9 (27.3%)
$Comb_{NoSmote}$	0.92	< 0.0001	> 91%	0.92	100.0	84.6	92.3	132 (80.0%)	132 (100.0%)	0 (0.0%)	33 (20.0%)	24 (72.7%)	9 (27.3%)
$Comb_{Smote}$	0.81	< 0.0001	> 89%	0.83	77.8	87.8	82.8	139 (84.2%)	137 (98.6%)	2 (1.4%)	26 (15.8%)	19 (73.1%)	7 (26.9%)

Abbreviations: AUC: Area under the Curve, Se: Sensitivity, Sp: Specificity, BAcc: Balanced Accuracy, APT: Acute Pulmonary Toxicity, Rad_NoSmote: Radiomics-Model without Smote, Rad_Smote: Radiomics-Model with Smote, Pmap_NoSmote: Pmap-Model without Smote, Pmap_Smote: Pmap-Model with Smote, Comb_NoSmote: Combined-Model without Smote, Comb_Smote: Combined-Model with Smote

Supplementary Table 5: Analysis of the each model's discrimination between patients with or without APT ≥ grade 3 in the testing set

Set	AUC	p	Cut-off	C-index	Se	Sp	BAcc	Number of patients, n (%)					
								Below the cutoff (Low risk of APT ≥ grade 3)			Above the cutoff (High risk of APT ≥ grade 3)		
								Total	Without APT	With APT	Total	Without APT	With APT
Rad$_{NoSmote}$	0.52	0.91	> 19%	0.65	100.0	30.0	65.0	30 (71.4%)	28 (93.3%)	2 (6.7%)	12 (28.6%)	12 (100.0 %)	0 (0.0%)
Rad$_{Smote}$	0.85	0.0001	> 25%	0.89	100.0	77.5	88.8	31 (73.8%)	31 (100.0%)	0 (0.0%)	11 (26.2%)	9 (81.8%)	2 (18.2%)
Pmap$_{NoSmote}$	0.86	< 0.0001	> 89%	0.90	100.0	80.0	90.0	32 (76.2%)	32 (100.0%)	0 (0.0%)	10 (28.8%)	8 (80.0%)	2 (20.0%)
Pmap$_{Smote}$	0.89	< 0.0001	> 86%	0.94	100.0	87.5	93.8	35 (83.3%)	35 (100.0%)	0 (0.0%)	7 (16.7%)	5 (71.4%)	2 (28.6%)
Comb$_{NoSmote}$	0.93	< 0.0001	> 91%	0.91	100.0	82.5	91.3	33 (78.6%)	33 (100.0%)	0 (0.0%)	9 (21.4%)	7 (77.8%)	2 (22.2%)
Comb$_{Smote}$	0.88	< 0.0001	> 89%	0.86	100.0	72.5	86.3	29 (69.0%)	29 (100.0%)	0 (0?0%)	13 (31.0%)	11 (84.6%)	2 (15.4%)

Abbreviations: AUC: Area under the Curve, Se: Sensitivity, Sp: Specificity, BAcc: Balanced Accuracy, APT: Acute Pulmonary Toxicity, Rad_NoSmote: Radiomics-Model without Smote, Rad_Smote: Radiomics-Model with Smote, Pmap_NoSmote: Pmap-Model without Smote, Pmap_Smote: Pmap-Model with Smote, Comb_NoSmote: Combined-Model without Smote, Comb_Smote: Combined-Model with Smote

Supplementary Figure 1: Overlap between the PmapRad and the Pmap maps

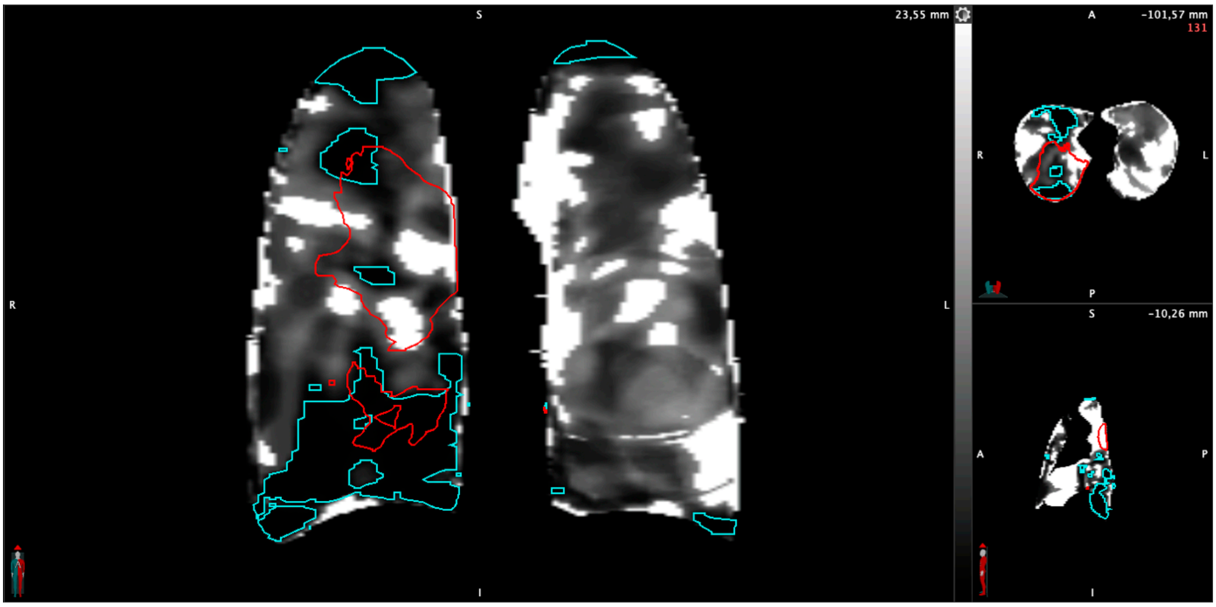

Caption: Red: Pmap region, blue: PmapRad region

Supplementary Figure 2: Comparison between each model in the testing sets based on the precision-recall curve for the prediction of APT ≥ grade 2

- a: Rad$_{NoSmote}$ model
- b: Rad$_{Smote}$ model
- c: Pmap$_{NoSmote}$ model
- d: Pmap$_{Smote}$ model
- e: Comb$_{NoSmote}$ model
- f: Comb$_{Smote}$ model

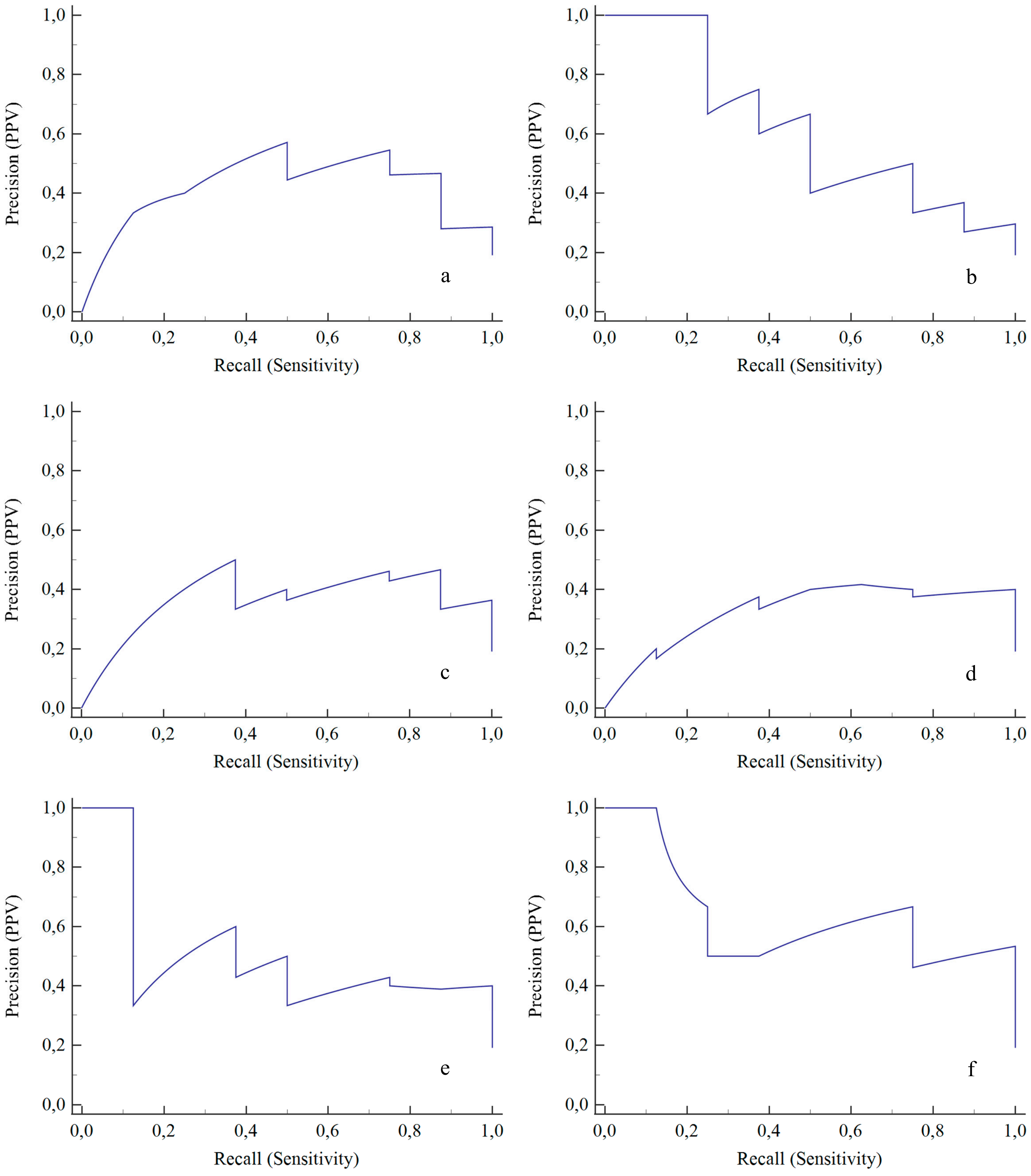

Supplementary Figure 3: Comparison between each model in the testing sets based on the calibration curve for the prediction of APT ≥ grade 2

- a: Rad$_{NoSmote}$ model
- b: Rad$_{Smote}$ model
- c: Pmap$_{NoSmote}$ model
- d: Pmap$_{Smote}$ model
- e: Comb$_{NoSmote}$ model
- f: Comb$_{Smote}$ model

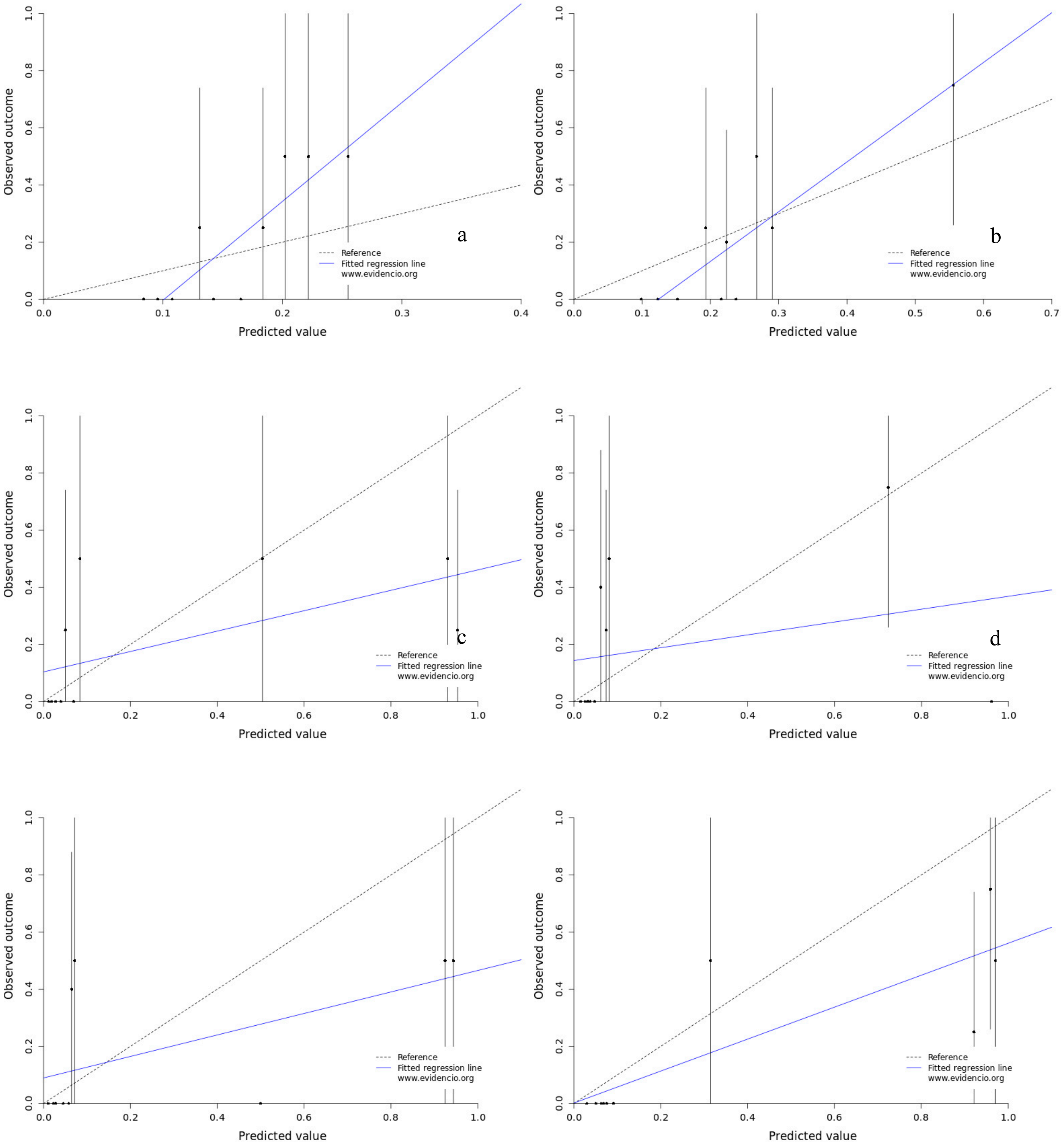

Supplementary Figure 4: Comparison between each model in the training (a) and testing (b) sets based on the ROC curve for the prediction of APT ≥ grade 3

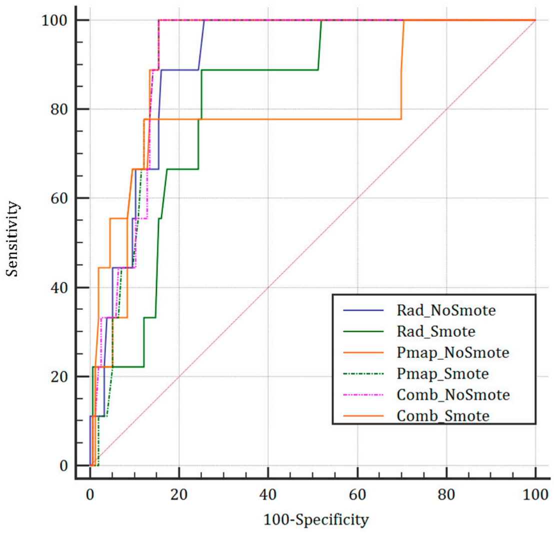

A

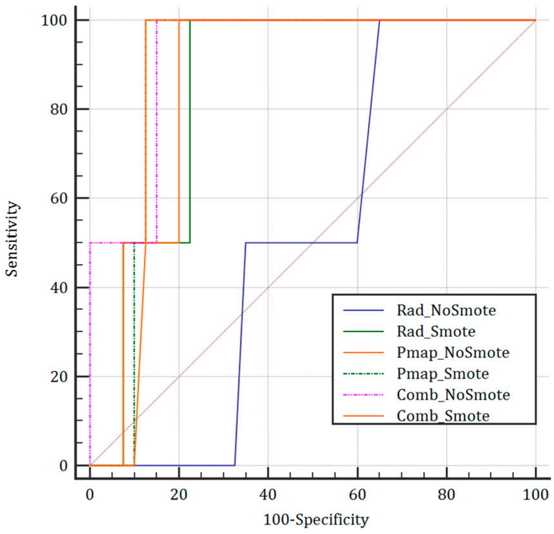

B

Abbreviation: APT: Acute Pulmonary Toxicity, Rad_NoSmote: Radiomics-Model without Smote, Rad_Smote: Radiomics-Model with Smote, Pmap_NoSmote: Pmap-Model without Smote, Pmap_Smote: Pmap-Model with Smote, Comb_NoSmote: Combined-Model without Smote, Comb_Smote: Combined-Model with Smote

Supplementary Figure 5: Comparison between each model in the training (a) and testing (b) sets based on the Decision Curve Analysis for the prediction of APT ≥ grade 3

A.

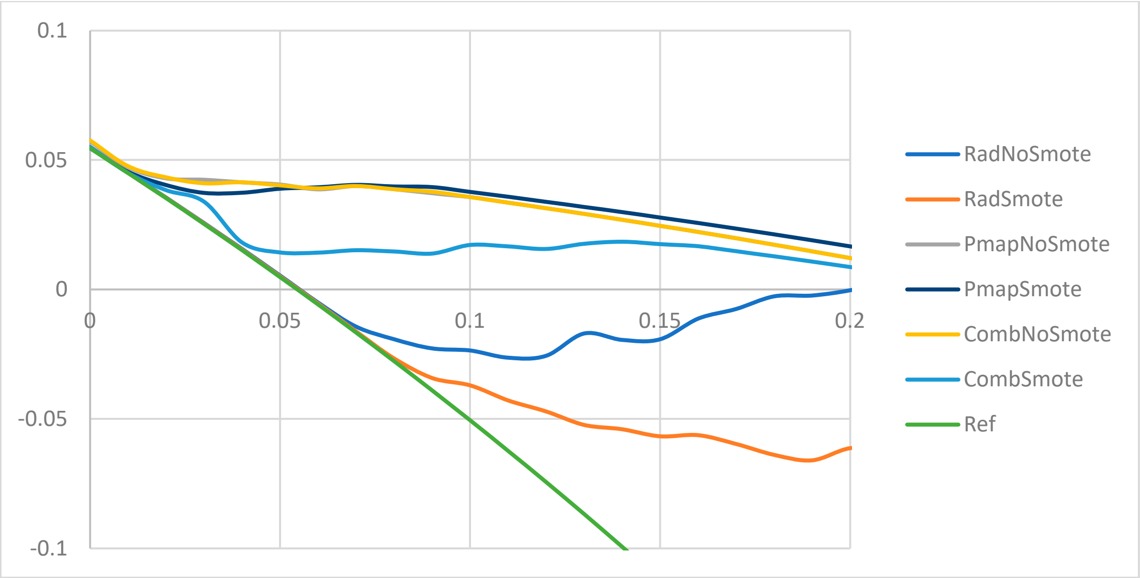

B.

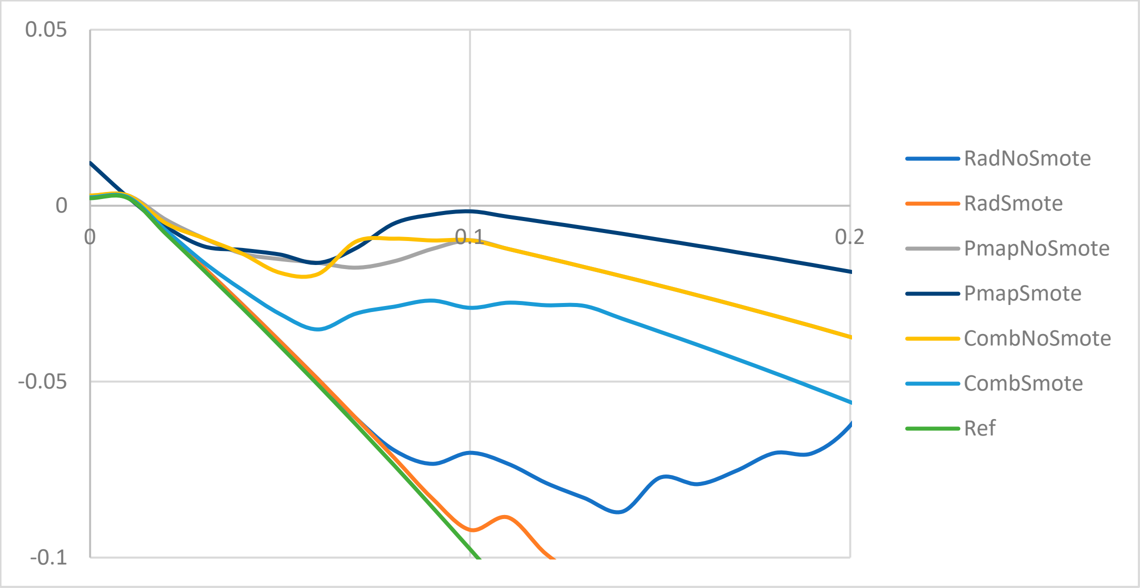

Abbreviation: APT: Acute Pulmonary Toxicity, Rad_NoSmote: Radiomics-Model without Smote, Rad_Smote: Radiomics-Model with Smote, Pmap_NoSmote: Pmap-Model without Smote, Pmap_Smote: Pmap-Model with Smote, Comb_NoSmote: Combined-Model without Smote, Comb_Smote: Combined-Model with Smote
